# Venereal Transmission of Vesicular Stomatitis Virus by *Culicoides sonorensis* Midges

**DOI:** 10.3390/pathogens9040316

**Published:** 2020-04-24

**Authors:** Paula Rozo-Lopez, Berlin Londono-Renteria, Barbara S. Drolet

**Affiliations:** 1 Department of Entomology, Vector Biology Laboratory, Kansas State University, Manhattan, KS 66506, USA; paularozo@ksu.edu; 2 United States Department of Agriculture, Agricultural Research Service, Arthropod-Borne Animal Diseases Research Unit, Manhattan, KS 66502, USA

**Keywords:** vesicular stomatitis virus, *Culicoides* midges, non-conventional transmission, venereal transmission, reproductive anatomy, mating behavior

## Abstract

*Culicoides sonorensis* biting midges are well-known agricultural pests and transmission vectors of arboviruses such as vesicular stomatitis virus (VSV). The epidemiology of VSV is complex and encompasses a broad range of vertebrate hosts, multiple routes of transmission, and diverse vector species. In temperate regions, viruses can overwinter in the absence of infected animals through unknown mechanisms, to reoccur the next year. Non-conventional routes for VSV vector transmission may help explain viral maintenance in midge populations during inter-epidemic periods and times of adverse conditions for bite transmission. In this study, we examined whether VSV could be transmitted venereally between male and female midges. Our results showed that VSV-infected females could venereally transmit virus to uninfected naïve males at a rate as high as 76.3% (RT-qPCR), 31.6% (virus isolation) during the third gonotrophic cycle. Additionally, VSV-infected males could venereally transmit virus to uninfected naïve females at a rate as high as 76.6% (RT-qPCR), 49.2% (virus isolation). Immunofluorescent staining of micro-dissected reproductive organs, immunochemical staining of midge histological sections, examination of internal reproductive organ morphology, and observations of mating behaviors were used to determine relevant anatomical sites for virus location and to hypothesize the potential mechanism for VSV transmission in *C. sonorensis* midges through copulation.

## 1. Introduction

Vesicular stomatitis virus (VSV) (Rhabdoviridae: *Vesiculovirus*) is a single-stranded, negative-sense, RNA pathogen responsible for vesicular stomatitis (VS) disease in cattle, horses, and swine [1]. VSV causes annual outbreaks in enzootic regions from northern South America to southern Mexico, infecting a large percentage of susceptible species [1]. In the U.S., VSV re-emerges sporadically with incursions originating from these southern enzootic regions moving northward into southwestern states at approximately 3 to 10-year intervals [1,2,3]. Epizootic viruses can overwinter, in an as yet identified natural reservoir, resulting in a second-year outbreak of the same viral genotype [3,4]. The epizootiology of VS is complex and comprises a wide variety of variables from a broad vertebrate host range, with variation in clinical outcome due to host species and site of initial infection, to the rapid transmission within animal herds by direct contact and fomites [1,5]. Furthermore, there is a diversity of suspected and potential transmission vector species acting as both mechanical and biological vectors throughout temperate and tropical ecosystems [6]. During VSV outbreaks in the U.S., *Culicoides* biting midges (Diptera: Ceratopogonidae) and *Simulium* black flies (Diptera: Simuliidae) have important roles in the initial introduction of VSV into animal herds and contribute to outbreak spread in the absence of animal movement [1,2,3,7]. Specifically, *Culicoides sonorensis* is one of the most common midge species associated with livestock agriculture [8,9] and a known biological transmission vector of VSV [10,11,12,13,14,15].

Transmission of VSV via *Culicoides* female bites is dependent upon available viremic hosts or infected hosts exhibiting skin-associated vesicular lesions containing large amounts of virus [16]. Blood feeding midges may acquire virus from blood [6], vesicular lesions, or from feeding on intact skin contaminated by vesicular fluid or virus-laden saliva [17,18]. However, the resulting pantropic systemic infection of *C. sonorensis* midges following oral ingestion of VSV [10], suggests that the interrelationships between the virus and vector may not be restricted to a bloodmeal-midgut-salivary gland-bloodmeal transmission route [6]. VSV infection and replication in reproductive tissues indicate that non-conventional routes of transmission might also occur. Specifically, VSV replication has been shown to occur in the ovarial epithelium and within the developing oocytes, suggesting that transovarial transmission might be possible [10]. Likewise, VSV infection of other relevant reproductive tissues and the rectal ampulla [10] suggests potential scenarios for trans-ovum transmission and transmission during sexual contact. Previously, VSV infection in *Culicoides* males has not been of interest because males were not believed to be involved in the transmission of viruses [19]. Since only females feed on blood, studies have been confined to the role females play in transmission and virus maintenance. However, in recent years, it has been suggested that males of some vector species might have a synergistic involvement in arbovirus transmission [20,21].

Therefore, determining the role of males, specifically the role venereal transmission (VNT) plays, in VSV maintenance in *Culicoides* populations, could lead to a more comprehensive understanding of 1) potential virus persistence in nature during interepidemic periods; 2) the overwintering of some viral genotypes leading to multi-year outbreaks; and 3) vector transmission dynamics during outbreaks. Herein, we report the first evidence for venereal transmission of any arbovirus in *Culicoides* spp. biting midges, and the first evidence for venereal transmission of VSV in any vector species. Additionally, we detail the mating behavior and morphological descriptions of *C. sonorensis* female and male reproductive anatomy with localization of VSV to provide insights into the potential mechanism of VNT.

## 2. Results

### 2.1. Venereal Transmission from Orally Infected C. sonorensis Females to Naïve Males

**Figure 1:**
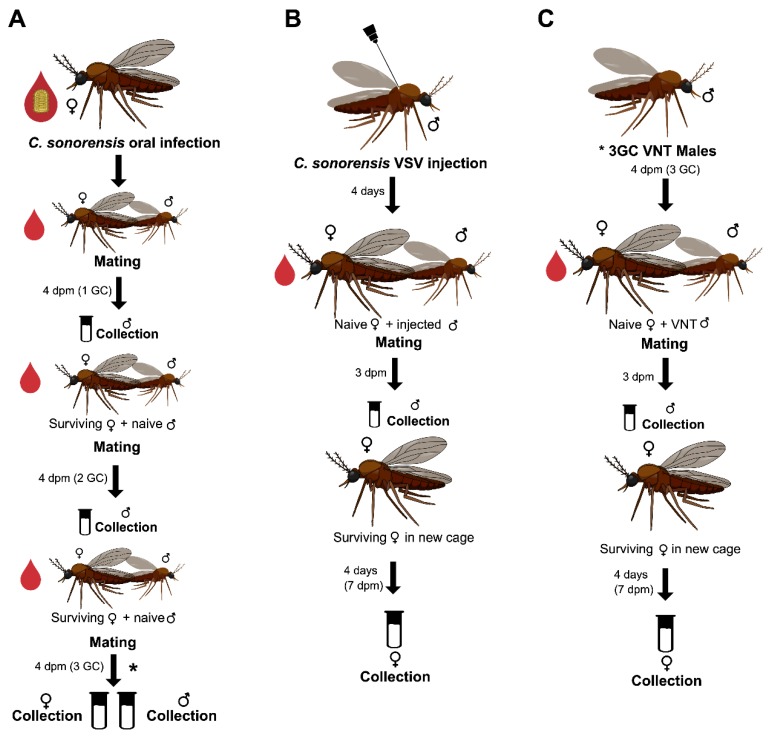
Experimental design to test venereal transmission of vesicular stomatitis virus (VSV) in *Culicoides sonorensis* midges. (**A**) Venereal transmission from VSV-fed females to naïve males. (**B**) Venereal transmission from intrathoracically VSV-injected males to naïve females. (**C**) Venereal transmission from 3rd gonotrophic cycle (GC)-mated males * (obtained in experiment (**A**)) to naïve females.

**Figure 2:**
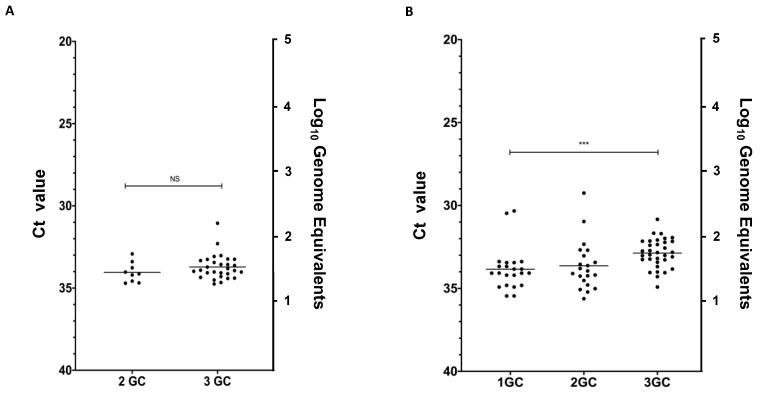
RT-qPCR cycle threshold (Ct) values (left Y-axis) and Log10 viral genome equivalents (right Y-axis) for individual VSV-positive (Ct ≤ 36) *C. sonorensis* midges. Non-parametric tests were used to compare distributions of Ct values between gonotrophic cycles. (**A**) *C. sonorensis* males infected with VSV following cohabitation with orally infected females at the end of the second (2 GC) and third (3 GC) gonotrophic cycles (*p* value = 0.196; NS, not significant). (**B**) *C. sonorensis* females orally infected with VSV tested at the end of each bloodmeal-induced gonotrophic cycle (1 GC, 2 GC, 3 GC) (*p*-value = 0.0002; *** *p* < 0.001).

Colonized *C. sonorensis* midges typically survive an average of 14–21 days depending on the number and types of manipulations to which they are subject. If provided blood meals and allowed to cohabitate with males, female survival rates are adequate to analyze three gonotrophic cycles. To determine rates of venereal transmission from infected females to age-matched naïve males, four mating experiments, two tested by RT-qPCR and two tested by virus isolation (VI), were conducted through three sequential bloodmeal-induced gonotrophic cycles (GC). Naïve adult males were made available for copulation by cohabiting with VSV-fed females from 0 to 4 days post-feeding (dpf) (1 GC), 4 to 8 dpf (2 GC), and 8 to 12 dpf (3 GC) (Figure 1A). All 88 surviving males collected at the end of 1 GC tested negative for viral RNA (Table 1). During the second GC, 15.2% and 20% of the surviving males paired with orally infected females tested positive for viral RNA by RT-qPCR and for infectious virus by cytopathic effects (CPE) in cell culture, respectively. Cycle threshold (Ct) values of males paired with 2 GC females ranged from 34.7 to 32.9 (10^1^ to 10^2^ genome equivalents) (Figure 2A). At the end of 3 GC, 31.6% of the males were CPE positive and 76.3% RT-qPCR positive (Table 1) with Ct values ranging from 34.7 to 31.1 (10^1^ to10^2^ genome equivalents) (Figure 2A). Additionally, orally infected females (10 to 20 from each of two mating experiment) were collected at the end of each bloodmeal-induced gonotrophic cycle to be tested by RT-qPCR (Figure 2B). Ct values for females collected at the end of 1 GC ranged from 35.4 to 30.3, 35.6 to 29.3 for 2 GC, and 34.9 to 30.8 for 3 GC.

### 2.2. Venereal Transmission of VSV from Intrathoracically Injected C. sonorensis Males to Naïve Females

**Figure 3:**
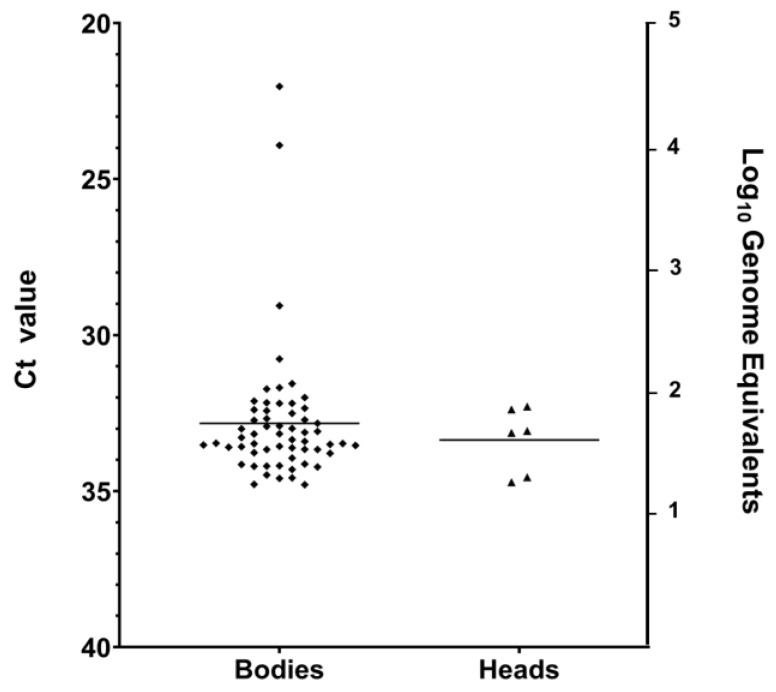
RT-qPCR cycle threshold (Ct) values (left Y-axis) and Log10 of viral genome equivalents (right Y-axis) for positive (Ct ≤ 36) *C. sonorensis* female bodies and heads infected with VSV following cohabitation with males infected by microinjection.

Mating experiments were conducted to determine whether males 4 days post-injection (dpi) can venereally transmit VSV to age-matched naïve adult females, as tested by RT-qPCR and CPE (Table 2, Figure 1B). Of females surviving 7 days after the first exposure to infected males (11–14 days post emergence for both males and females), 49.2% were CPE positive (Table 2). To determine if the virus acquired by venereal transmission could disseminate into the salivary glands of females, we separately tested bodies (n = 77) and then tested heads with glands from RT-qPCR-positive bodies (n = 59). The bodies of 76.6% of the females tested positive for viral RNA (Table 2) with Ct values ranging from 34.8 to 22 (10^1^ to 10^5^ genome equivalents) (Figure 3). The heads of 11.63% of the females tested positive for viral RNA (Table 2) with Ct values ranging from 34.8 to 32.3 (10^1^ to 10^2^ genome equivalents) (Figure 3). Additionally, a sub-sample of the inoculated males was tested at 4 dpi (when introduced into the mating cages) and at 7 dpi (when removed from mating cages) for the presence of VSV RNA (N = 5) and infectious virus (N = 5). All tested males were positive, with titers ranging from 1.35 × 10^5^ to 2.8 × 10^5^ PFU/mL by plaque assay.

### 2.3. Venereal Transmission of VSV from Venereally Infected C. sonorensis Males to Naïve Females

**Figure 4:**
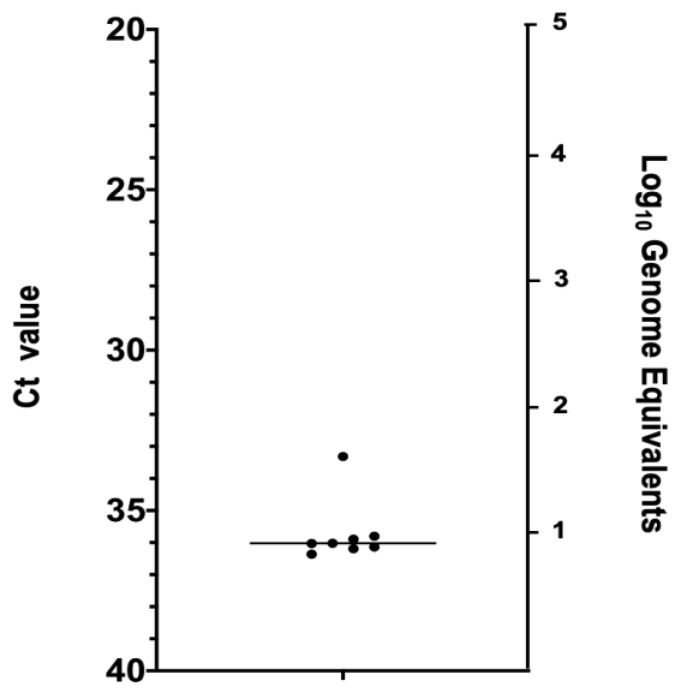
RT-qPCR cycle threshold (Ct) values (left Y-axis) and Log10 of viral genome equivalents (right Y-axis) for positive (Ct ≤ 36) *C. sonorensis* female bodies infected with VSV following cohabitation with venereally infected males.

Mating experiments were conducted to test venereal transmission of VSV from venereally infected males to younger naïve females (Figure 1C). Males used in these experiments had cohabitated with orally infected females during the third blood meal-induced gonotrophic cycle (3 GC). Younger females were used instead of age-matched in order to increase the chance of female survival at 7 days post mating. Of the 84 surviving females at 7 days post mating, 9.5% tested positive by RT-qPCR (Table 3) with Ct values ranging from 36.3 to 33.2 (10^1^ to 10^2^ genome equivalents) (Figure 4).

### 2.4. VSV Infection in Reproductive Tracts

We conducted three trials using VSV-immunofluorescent staining of reproductive organs of intrathoracically inoculated males and females to establish tissue tropism for VSV in the reproductive tract of midges at 4 dpi. Initially, virgin females were used; however, the underdeveloped ovary morphology did not allow visualization of precise locations for VSV. Consequently, to add clarity to the virus location within developing oocytes, mated and blood-fed females were used in all subsequent trials.

**Figure 5:**
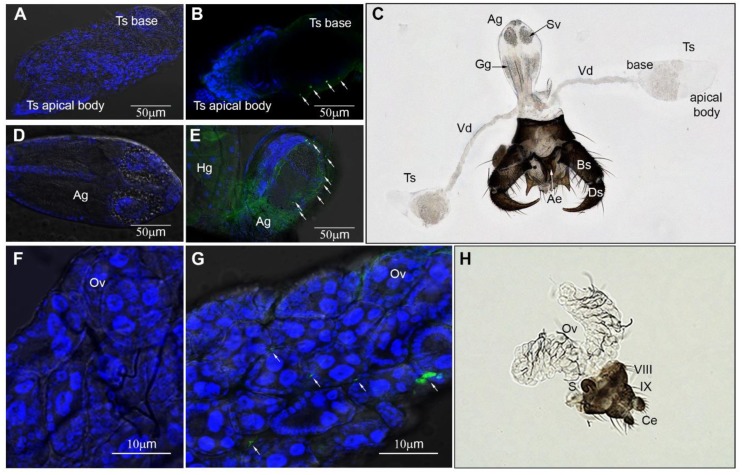
Immunofluorescent VSV-staining of intrathoracically infected *C. sonorensis*. (**A**) Testis (Ts) and (**D**) accessory gland (Ag) dissected from non-infected (negative control) males. (**B**) Testis (Ts) and (**E**) accessory gland (Ag) dissected from males 4 days post inoculation. Arrows denote VSV-positive staining (FITC-green puncta) in the epithelial layer of the testis base and outer epithelial layer of the Ag. Cellular nuclei were stained with DAPI (blue). (**C**) Male reproductive anatomy (brightfield 200×). Abbreviations: Ae: aedeagus, Ag: accessory gland, Bs: basistyle, Ds: dististyle, Gg: glutinous gland, Sv: seminal vesicle, Ts: testis, Vd: vas deferens. (**F**) Ovaries (Ov) dissected from non-infected (negative control) females. (**G**) Ovaries (Ov) dissected from females 4 days post inoculation. Arrows denote VSV-positive staining (FITC-green puncta) with DAPI nuclear stain (blue). (**H**) Female reproductive anatomy (brightfield 200×). Abbreviations: VIII: 8th abdominal segment, IX: 9th abdominal segment, Ce: cerci, Ov: ovary, S: spermatheca.

The VSV-positive fluorescent puncta in males (Figure 5B,E, Table 4) indicated viral infection of the epithelial layer at the base of the testes (91.7%), in the outer epithelial surface of the accessory gland (58.3%), and throughout tissues in the hindgut and the rectal region (100%). In contrast, vas deferens, ejaculatory duct, and terminalia did not show positive fluorescence.

**Figure 6:**
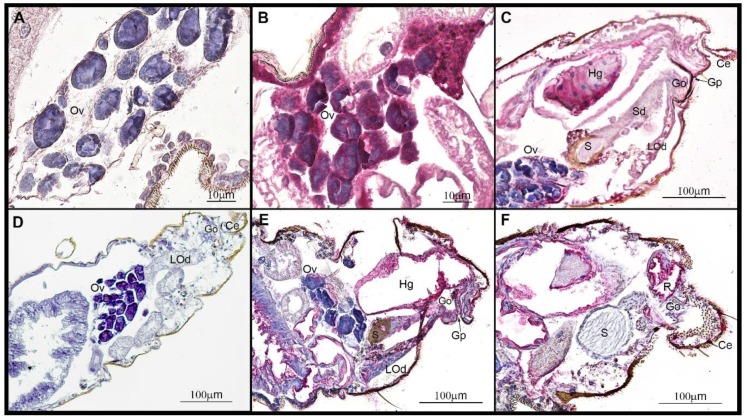
Immunohistochemical VSV-staining of orally infected *C. sonorensis* females. (**A**) Ovaries (Ov) and (**D**) abdomen of non-infected (negative control) females. (**B**) Ovaries (Ov) and (**C**,**E**,**F**) abdominal sections from females 9–13 days post feeding with VSV-positive antigen staining of viral nucleocapsid in red and counterstained with hematoxylin (blue). (**B**) VSV antigen staining in the ovarial sheaths and trachea. (**C**) VSV antigen staining in hindgut (Hg), gonopore (Gp), gonotreme (Go), lateral oviduct (LOd), spermathecal duct (Sd), and ovaries (Ov). (**E**) VSV antigen staining in hindgut (Hg), gonopore (Gp), gonotreme (Go), and lateral oviduct (LOd), with negative ovaries (Ov) and spermatheca (S). (**F**) VSV antigen staining in rectum (R) and gonotreme (Go), with negative spermatheca (S).

The intensity of positive fluorescence puncta in whole reproductive organs of females (Figure 5G, Table 4) was detected in the tracheal branches located in the space between ovarian sheaths (91.7%) and throughout tissues in the hindgut and the rectal region (100%). There were no apparent positive puncta in the spermatheca and spermathecal gland. Due to the inability to get intact whole reproductive tracts with undamaged ducts, we examined sagittal sections from 19 sequentially sampled virus fed females (9–13 dpf, time corresponding to 3 GC) used to first describe the temporal and spatial progression of VSV infection in *Culicoides* [10]. The positive staining (Figure 6B,C,E,F, Table 5) was detected in the ovaries (Ov) (36.8%), oviduct (Od) (16.8%), spermathecal duct (Sd) (26.3%), gonotreme (Go) and gonopore (Gp) (57.8%), and throughout tissues in the hindgut (Hg) and the rectal region (R).

### 2.5. Behavioral Observations of C. sonorensis Copulation

There is little information on the mating behavior, comparative function of the sex organs during mating, and the timing for efficient sperm transmission in *Culicoides* midges. In order to better understand the mating behavior of *C. sonorensis* and provide insight into the mechanism of VSV venereal transfer, we conducted observations of midge-matings under laboratory conditions.

*C. sonorensis* copulation occurred on the bottom of the cages without a swarming flight. When specimens of both sexes were introduced at 1 to 3 days post-emergence, they often rested for long periods with frequent antennal and wing movements. Mating attempts were initiated by males following the females with rapid walking movements and continual antennal vibrations culminating with the efforts of the male to climb onto the back of the female. If a blood meal was not offered, most females showed resistance behavior when males approached. The resistance behavior consisted of the females running rapidly, kicking males with their hind legs, and anteriorly curving the dorsal segments of the abdomen to avoid contact with the male claspers. However, 10–15 min after a blood meal was offered, fed females were receptive to the multiple attempts of males to establish genital contact. Males approached the female terminalia with curved abdomens and open claspers. After attachment, males would then rotate around the female until they were positioned 180° to the female. *Culicoides nubeculosus* also presents this 180° torsion, but for other species only a gentle torsion has been reported [22]. The genital contact (attachment) time was 420 ± 15 s. Detachment following copulation occurred rapidly by the claspers opening while the female pushed with their hind legs until separation.

From our observations in the laboratory, the presence of males stimulates female blood feeding, and subsequently, blood ingestion incites copulation. Moreover, *C. sonorensis* can repeatedly mate within each gonotrophic cycle, as previously reported for the variipennis complex (which includes *C. sonorensis*) [22]. Together these behaviors may impact VSV epidemiology by increasing viral exposure opportunities by blood-feeding females. Furthermore, the relatively long copulation time, and the possibility of multiple matings in a lifetime, makes the implications of VSV venereal transmission from females to males and males to females more likely to impact viral maintenance in *C. sonorensis* midge populations.

### 2.6. Anatomical Descriptions of C. sonorensis Male Reproductive Tract

Little information exists in the literature relative to the internal anatomy of males of the family Ceratopogonidae. Thus, to better understand how the venereal transmission of VSV is occurring from *C. sonorensis* males to females, we describe our morphological and anatomical observations of the male reproductive tract.

**Figure 7:**
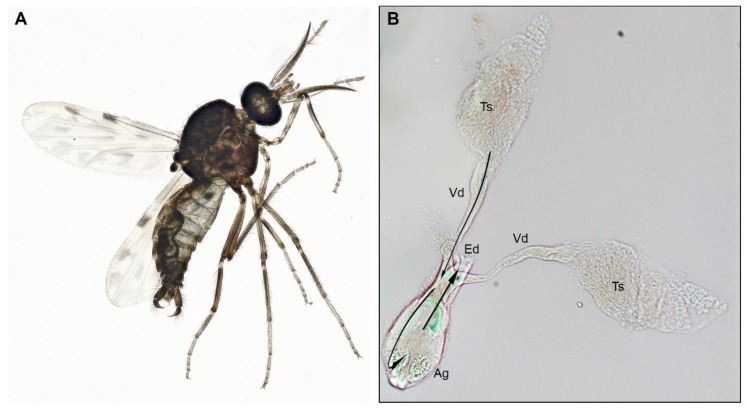
Anatomy of *Culicoides sonorensis* male (**A**) External male morphology. (**B**) Male reproductive tract detached from the terminalia. Arrows indicate sperm ejaculation pathway. Abbreviations: Ag: accessory gland, Ed: ejaculatory duct, Ts: testis, Vd: vas deferens.

The outer male abdomen is slender with the 9th segment, the tergum, and sternum fused in the shape of a sclerotized ring to which a prominent terminalia is attached (Figure 5C and Figure 7A). Two distinct sclerotized curved claspers are formed by a basal basistyle (Bs) and a claw-like apical dististyle (Ds) (Figure 5C). The aedeagus (Ae) is Y-shape and is held by a sclerotized structure on the ventral side (Figure 5C). The testes (Ts) are elongated pyriform concerted to a tubular vas deferens (Vd) (Figure 5A,B and Figure 7B). Each vas deferens is connected to the base of the accessory gland (Ag) (Figure 5C and Figure 7B) and directs the sperm through to the distal portion of the Ag which contains two circular seminal vesicles (Sv) on each side (Figure 5C and Figure 7B). Each Sv is surrounded by a layer of large secretory cells. Below each seminal vesicle is a pair of broadly ovoid glutinous glands (Gg) (Figure 5C and Figure 7B), which in other *Culicoides* species are known to contain secretory cells [23,24]. The spermatozoa and ejaculatory secretions (most likely transferred to a female in a spermatophore as reported in *Culicoides nubeculosus* [25] and *Culicoides melleus* [22,23]) are released through a common ejaculatory duct (Ed) at the base of the accessory gland which is connected to the aedeagus (Figure 7B).

The sperm transferred during mating consists of a mix of proteins, lipids, carbohydrates, salts, and steroid hormones produced in the male accessory glands, and possibly in the testes [25]. Despite the potential impact that this ejaculatory complex may have on the reproductive physiology and behavior of females, the sperm ejaculation route (Figure 7B) contributes to VSV maintenance in midge populations by allowing the efficient transmission of virus particles from the male into the female upon copulation.

### 2.7. Anatomical Descriptions of C. sonorensis Female Reproductive Tract

Venereal transmission from females to males has rarely been reported in the literature [26], mainly because the male produces all of the secretions that are exchanged during copulation. To better understand how the venereal transmission of VSV is occurring from *C. sonorensis* females to males, we describe the morphology and anatomy of the female reproductive tract.

**Figure 8:**
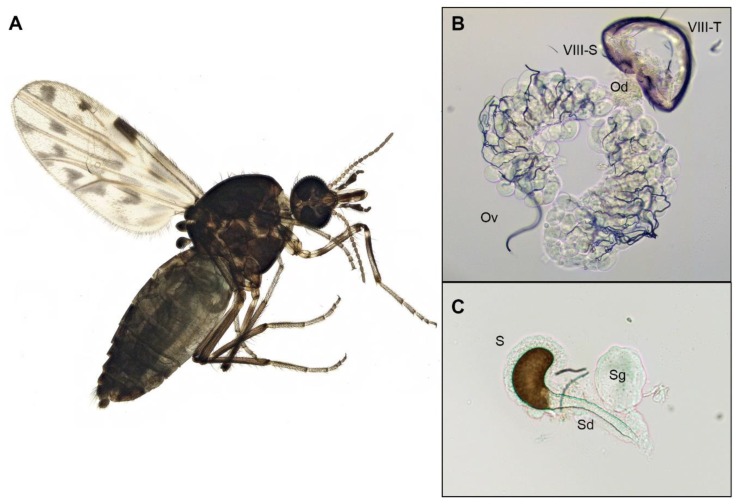
Anatomy of *Culicoides sonorensis* female (**A**) External female morphology. (**B**) Ovaries (Ov) joined by common oviduct (Od) attached to the 8th sternum (VIII-S). (**C**) Detailed portion of the spermatheca (S), spermathecal gland (Sg), and spermatheca duct (Sd).

The outer morphology of the female reproductive system is relatively simple, with a stout abdomen ending in a pair of small rounded cerci (Ce) with long sensory hairs visible below the 9th tergum (IX) (Figure 5H and Figure 8A). Internally, the female reproductive system is complex, presenting two ovaries (Ov) located at the anterior end of the female’s abdomen, usually internally located between the 5th and the 6th abdominal segments (Figure 5H and Figure 8B). The ovaries contain oocytes at similar stages of development. Each oocyte is surrounded by follicular cells and contain 3 to 5 nurse cells. The oocytes are held together by an epithelial sheath surrounded by a network of fine, branching tracheae. At the base of each ovary, there is a lateral oviduct (LOd) that fuses into a common oviduct (Od) which is attached to the 8th sternum (Figure 8B). The common oviduct posteriorly enlarges to a gonotreme (Go), which receives the sperm during copulation. The posterior end of the gonotreme is bifurcated into a hyaline duct known as the spermathecal duct (Sd), which contains a minute globular spermathecal gland (Sg) (also known as a female accessory gland) and ending in one sclerotized mushroom-shaped (convex or campanulate) spermatheca (S) (Figure 5H and Figure 8C) for sperm storage. The last section of the posterior end of the gonotreme, located in close proximity to the rectum (R), exits the female reproductive tract at the distal end of the female terminalia, the gonopore (Gp), which also serves to oviposit eggs. The polyandric behavior observed in *C. sonorensis* females, combined by the rich virus load in the anal region and gonopore, contribute to maintenance of VSV in midge populations by favoring virus transmission from females to multiple males in a lifetime of copulation events.

## 3. Discussion

Vesicular stomatitis outbreaks in temperate regions peak during summer and fall and typically stop after the first hard freeze, corresponding with the decrease in the number of vectors in affected areas [2]. Outbreak viruses can overwinter with the same viral genotype re-emerging for a second-year outbreak [3,27], as occurred in 2004–2005, 2005–2006, 2014–1015, and in the most recent 2019–2020 outbreaks. Several hypotheses have been proposed to explain the maintenance of VSV during inter-epidemic periods, mainly by suggesting the presence of a yet to be identified, natural mammalian reservoir [28,29]. However, from a vector perspective, the vertical and venereal transmission of arboviruses are possible maintenance mechanisms during inter-epidemic periods in which the virus is maintained in a vector population independent of feeding on viremic animals [30]. Among hematophagous Diptera, the venereal transmission of viruses of human and veterinary importance has been observed with bunyaviruses [31,32,33], flaviviruses [34,35,36,37,38,39,40,41], rhabdoviruses [42,43], and togaviruses [44,45] in mosquitoes and sand flies. However, no previous studies have reported venereal transmission by *Culicoides* biting midges for any arbovirus, nor for any insect species with VSV.

In this study, we have demonstrated the presence of VSV RNA and infectious virus in previously uninfected midges of both sexes following cohabitation with VSV-infected mates. Our study also revealed the location of viral antigen in the reproductive tracts of both males and females. For the first time, VSV infection of female *C. sonorensis* midges has been shown to occur not only during blood feeding but also during copulation. Venereal transmission of VSV from orally infected females to naïve males and from venereally infected males to naïve females suggests the virus could be maintained in midge populations at a low threshold during inter-epidemic periods and then reinitiate an outbreak when conditions for bite transmission are once again ideal. The testing of heads from positive VNT females was used as an indication of both dissemination and transmission potential. Although virus detected in the heads would include infected neural and optic tissues, previous in situ hybridization staining of infected midges showing significant VSV replication in salivary gland epithelium, and immunohistochemical staining showing VSV in the lumen of salivary glands ready for excretion [10], strongly suggest positive midge heads correlate to bite transmission potential.

Venereal transmission of VSV from females to males was shown to occur at higher rates during the females third gonotrophic cycle (8 to 12 days post-infectious feeding). This particular timing might be explained by the addition of subsequent blood meals which induce midgut expansion, increasing the number of midgut basal lamina micro-perforations and enhancing the likelihood of virus dissemination [46] as well as the stretching of follicular epithelial cells that facilitate higher viral infection rates of reproductive tracts [36,47].

The VSV-positive staining in female reproductive tracts suggests the mechanism of sexual transmission from infected females to males occurs by transmission of virus particles located at the distal end of the female terminalia (gonopore and rectum) upon contact during copulation. The VSV-positive fluorescent puncta in male reproductive tracts suggests the mechanism of sexual transmission of VSV from infected males to females likely follows the sperm ejaculation pathway in which virus particles are released from the base of the testis into the accessory gland where the spermatozoa are mixed with ejaculatory secretions passing through the ejaculatory duct and are released into the female reproductive tract upon copulation.

It has been demonstrated in mosquitoes that males and copulation activities have a synergistic effect on transmission by influencing the female vectorial capacity [20,48]. To date, the role of male midges in the transmission dynamics of VSV or other midge-transmitted viruses has not been reported. Our results of venereal VSV transmission between males and females suggests a potentially important role for males in the natural survival and maintenance of interepidemic VSV. Although male midges are not hematophagous, our study shows they can acquire virus and become infected after copulation with infected females and then transmit VSV to naïve females during subsequent mating. From the ecological and epidemiological perspective, males not only contribute to the overall virus overwintering and transmission, but from our behavioral observations, they also increase the percentage of females that successfully blood feed.

A caveat to mating midges in cages requires the consideration that VSV could have been transmitted between the sexes not only during mating but also through other types of contact with salivary or anal secretions. To undoubtedly determine if infection occurred during the act of mating, we tested if VSV could be transmitted when an induced mating technique was used [49]. This way, only a brief contact of the genitalia would be allowed. Unfortunately, successful copulation was not achieved due to the complex *C. sonorensis* mating physiology and the long duration of attachment required for successful copulation, which is possibly due to spermatophore formation and transfer observed in other *Culicoides* species [23,24].

While there are limitations to confined laboratory experiments in induced mating, our research shows VSV midge-to-midge transmission after cohabitation with orally infected, microinjected, or venereally infected midges of the opposite sex. Our description of *C. sonorensis* mating behavior and the morphological descriptions of the internal reproductive systems of both sexes extends the knowledge of *Culicoides* midges and relates to previous studies on *Culicoides melleus* [23,24,50] and *Culicoides nubeculosus* [51]. This research shows the importance of males in VSV transmission dynamics and in the maintenance of VSV in nature. Additionally, the significant VSV-positive staining of female reproductive tissues suggests vertical transmission may also play a role in VSV maintenance. While further studies are needed to determine the effects of VSV vertical transmission, venereal transmission to oviposition, mating behavior, and mate choices of infected/uninfected midges, these results highlight the need to incorporate alternative routes of transmission in understanding arbovirus outbreaks.

## 4. Materials and Methods

### 4.1. Virus and Cells

The New Jersey serotype of VSV (1982 bovine field isolate) was grown in porcine epithelial cells (AG08113; Coriell Institute, Camden, NJ, USA)) in Eagles MEM with Earle’s salts (Sigma, St. Louis, MO, USA) and 199E Media (2% FBS, 100U penicillin/streptomycin sulfate) at 37 °C with 5% CO_2_. Vero MARU cells (VM; Middle America Research Unit, Panama, Panama) grown in 199E media at 37 °C with 5% CO_2_ were used to detect and titer infectious virus in midge samples by standard plaque assay.

### 4.2. VSV Infection of Culicoides sonorensis Midges

Adult *C. sonorensis* midges used were from the AK colony maintained by USDA, Agricultural Research Service, Arthropod-Borne Animal Diseases Research Unit at the Center for Grain and Animal Health Research in Manhattan, KS, USA. Midges were reared as previously described [52]. Virgin female *C. sonorensis* midges (1–3 days post emergence) were allowed to feed on a glass, 37 °C water-jacketed bell feeder with a parafilm membrane/cage interface for 60 min. The VSV-blood meal consisted of defibrinated sheep blood (Lampire Biological Products, Pipersville, PA, USA) containing 4.25 × 10^8^ PFU VSV-NJ. Fully engorged blood-fed females were sorted from unfed and partially fed females and placed in cardboard maintenance cages. For positive controls, 1–3 day post emergence virgin midges were anesthetized with CO_2_, intrathoracically inoculated with VSV-NJ, and placed in maintenance cages. Intrathoracic injections were made dorsally at the prescutellar area using a volume of 46 nl (1.4 × 10^4^ PFU) for males and 60 nl (1.8 × 10^4^ PFU) for females using a Nanoject II injector (Drummond Scientific Company, Broomall, PA, USA). Injected volumes were determined as the maximum capacity for the male and female body size. Adult midges were maintained in environmental chambers at 25 ± 1 °C and 80% relative humidity with a 13:11 light: dark cycle and offered 10% sucrose solution ad libitum.

### 4.3. Venereal Transmission Assays

Venereal transmission of VSV from infected females to naïve males was tested (Figure 1A) for each of the three-blood meal-induced gonotrophic cycles (1–3GC). VSV-blood-fed virgin females were placed in cages with age-matched naïve males at a ratio of 2:1 females to males. Four days post-mating (dpm), all surviving males were individually collected in 300 µL of TRIzol (Invitrogen; Thermo Fisher Scientific, Inc., Waltham, MA, USA) for RT-qPCR testing, or in 500 µL of antibiotic medium (199E cell culture medium, 200 U/mL penicillin, 200 μg/mL streptomycin, 100 μg/mL gentamycin, and 5 μg/mL amphotericin B) for plaque assays and CPE. Collected midges were stored at −80 °C until further processing. Surviving females were moved to new cages and age-matched naïve males were again added at a ratio of 2:1. Midges were offered non-infectious blood meals for 60 min at the start of each cohabitation to initiate a gonotrophic cycle.

To test venereal transmission of VSV from infected males to naïve females (Figure 1B), four days post intrathoracic injection (dpi), males were transferred to a cage containing age-matched naïve females in a ratio of 2:1 females to males. Midges were offered a non-infectious blood meal for 60 min to initiate a gonotrophic cycle. Following three days of cohabitation, all surviving males were collected as above, and all surviving females were transferred to a new cage and kept for an additional four days. Seven days after cohabitation/mating (7 dpm), all surviving females were collected as above. To determine the VSV titer in males during the cohabitation period, a subsample of the infected males, 4 dpi and 7 dpi, was tested for virus by plaque assay.

To test venereal transmission of VSV from venereally infected males to naïve females (Figure 1C), we used surviving males at the end of the cohabitation period with orally infected females at the third blood meal-induced gonotrophic cycle. Venereally infected males were transferred to a cage containing naïve females in a ratio of 2:1 females to males. Midges were offered a non-infectious blood meal for 60 min to initiate a gonotrophic cycle. Following three days of cohabitation, all surviving males were collected as above, and all surviving females were transferred to a new cage and kept for an additional four days. Seven days after initial cohabitation, all surviving females were individually collected in 300 µL of TRIzol for RT-qPCR testing.

### 4.4. RNA Extraction and RT-qPCR for VSV Detection

Frozen TRIzol midge samples were thawed on ice and homogenized by high-speed shaking with a Bead Mill Homogenizer (Omni, Kennesaw, GA, USA) for 2 min at 3.1 m/s. Samples were centrifuged at 12,000× *g* for 6 min to pellet the debris. Total RNA was extracted using Trizol-BCP (1-bromo-3chloropropane; ThermoFisher Life Technologies, Waltham, MA). RNA was precipitated using isopropanol, washed in 75% ethanol, and eluted in 50 μL of nuclease-free water. RNA extracts were analyzed using TaqMan Fast Virus 1-Step MasterMix (Applied Biosystems; Thermo Fisher Scientific, Inc., Waltham, MA, USA) in a RT-qPCR targeting the L segment [53]: forward primer VSVNJ7274: 5′-TGATTCAATATAATTATTTTGGGAC-3; reverse primer VSVNJ7495: 5′-AGG CTCAGAGGCATGTTCAT-3′; probe: FAM-TTGCACACCAGAACATTCAA-3′-BHQ1. For amplification, the following temperature profile was used: Reverse-transcription 1 cycle at 50 °C for 5 min, denaturing and polymerase activation at 95 °C for 20 s, and amplification: 40 cycles of 95 °C for 15 s and 60 °C for 60 s. Samples were initially tested in pools containing RNA of 5 individual midges followed by testing of individual samples from positive pools to determine the exact number of positive individuals. Based on Ct values reported for VNT of dengue, Zika, and chikungunya viruses in mosquitoes [36,37,41,45], RT-qPCR reactions with Ct ≤ 36 were considered positive for VSV RNA. Additionally, to determine if virus acquired by venereal transmission could disseminate into salivary glands of females, pools of bodies and heads (containing the proximal region of the salivary glands) were assayed separately. Subsequent testing of individual bodies and heads from positive pools was conducted to determine the exact number of positive samples.

Standard curves and calculation of Ct values were carried out with the 7500 Fast Dx software (Applied Biosystems; Thermo Fisher Scientific, Inc., Waltham, MA, USA). Ct values were plotted against the log of VSV genome equivalents. The linear regression (y = −3.30578x + 11.02683) was used to determine the amount of viral genomic ssRNA per midge. Genome equivalents were calculated with the published VSV genome molecular weight [54] and the NEBioCalulator (https://nebiocalculator.neb.com/#!/ssrnaamt).

### 4.5. Cytopathic Effect and Plaque Assays

To isolate infectious virus, frozen midges stored in 500 μL antibiotic media were thawed on ice and individually homogenized as above. Samples were centrifuged at 12,000× *g* for 6 min to pellet debris. A subsample of cleared supernatant (200 μL) was added to a monolayer of VM cells in a 24-well plate and incubated at 37 °C for seven days at 5% CO_2_. Observation of cytopathic effects (CPE) after one or two passages were used as an indicator of infectious virus within that sample. All CPE+ wells were confirmed as VSV+ by testing for viral RNA by RT-qPCR, as described above. All homogenates with positive CPE at the first passage were further analyzed to determine infectious virus titer by standard plaque assay inoculating 200 μL of the original cleared supernatant sample on VM cells in 6-well plates and incubating at 37 °C for three days at 5% CO_2_.

### 4.6. Statistical Analysis

Data were pooled from independent replicates of each experiment. Statistical methods were not used to predetermine the sample size. The proportion of infected midges was calculated by dividing the number of infected whole midges by the total number of midges tested. A female with virus found in the body but not in the head was considered as a non-disseminated infection. When the virus was found in both the body and the head, the midge was determined to have a disseminated infection. The proportion of females with disseminated infection was calculated as the number of midges with positive heads divided by the total number of infected midges. Non-parametric tests were used to compare Ct distributions between gonotrophic cycles. GraphPad Prism version 8 (GraphPad Software Inc., La Jolla, CA, USA) was used for statistical analysis and creation of graphs.

### 4.7. Fluorescent Immune Assay and Immunohistochemistry

To determine VSV localization in the reproductive tissues of infected *C. sonorensis* midges that might allow VNT, intrathoracically injected males and females (4 dpi) were CO_2_ anesthetized and reproductive tracts were dissected in PBST (PBS + 0.5% of Triton X 100; PH 7.4). Tissues were fixed in 4% paraformaldehyde for 4 h and washed in PBST. All reproductive tracts were blocked in 1% Normal Goat Serum for one hour and sequentially incubated with rabbit anti-VSV-NJ nucleocapsid protein antibody (dilution 1:500 or 1:1000) at room temperature (RT). After a 1-day incubation, the reproductive tracts were washed in PBST for 4 h. Binding of primary antibodies was detected by incubating tissues with 1:300 or 1:500 dilution of Alexa Fluor 488 IgG Alpaca anti-Rabbit (Jackson Immuno Research, West Grove, PA, USA). Following a 4 h incubation in the dark at RT, samples were washed in PBST for 4 h. Cell nuclei were stained with 10 ug/mL of DAPI (40,6-diamidino-2-phenylindole) (Invitrogen; Thermo Fisher Scientific, Inc., Waltham, MA, USA). Reproductive tracts were mounted on slides using 100% glycerol and examined in confocal microscope LSM700 (Zeiss International, Oberkochen, Germany). Images were captured using ZEN software (Zeiss International, Oberkochen, Germany). Reproductive tracts of non-injected midges were treated similarly and served as negative controls.

To better determine the virus localization in the reproductive organs of orally infected *C. sonorensis* females, sagittal sections from sequentially sampled VSV-fed females (9–13 dpf) from a previous study [10] were examined and captured using an All-in-One Fluorescence Microscope (BZ-X810; Keyence Corporation, Itasca, IL, USA).

### 4.8. Behavioral Observations of C. sonorensis Copulation

All assays were conducted in round carton cages of 9 cm diameter and 5 cm height. Observations of mating were made using a Nikon SMZ-1500 binocular stereo zoom microscope (Nikon Instruments Inc., Melville, NY, USA). All observations were carried out under laboratory conditions (25 ± 2 °C, 65% RH).

## Figures and Tables

**Table 1:** Venereal transmission rates from orally infected females to naïve non-infected males during three gonotrophic cycles (GC) as detected by whole-body RT-qPCR or CPE.

GC	Initial Number of Midges	Surviving ♂ (%) ^1^	VSV RT-qPCR+ ♂ (%) ^2^	VSV CPE+ ♂ (%) ^3^
1	240 ♀ and 120 ♂	88/120 (73.3%)	0	ND
2	199 ♀ and 100 ♂	59/100 (59%)	9/59 (15.2%)	ND
2	104 ♀ and 52 ♂	30/52 (57.7%)	ND	6/30 (20%)
3	131 ♀ and 65 ♂	38/65 (58.5%)	29/38 (76.3%)	ND
3	64 ♀ and 32 ♂	19/32 (59.4%)	ND	6/19 (31.6%)

^1^ Surviving males were sampled 4 days after initial cohabitation, which was determined as the end of each blood meal-induced gonotrophic cycle (GC). ^2^ Vesicular stomatitis virus (VSV)-positive males detected by RT-qPCR. ^3^ VSV-positive males detected by cytopathic effect (CPE). ND, not determined.

**Table 2:** Venereal transmission from VSV-injected *Culicoides* males (4 dpi) to age-matched naïve females as detected by RT-qPCR of individual bodies and heads and CPE of whole midges.

Initial Number of Midges	Surviving ♀ (%) ^1^	VSV RT-qPCR+ ♀ Bodies (%)	VSV RT-qPCR+ ♀ Heads (%)	VSV CPE+ Whole ♀ (%)
58 ♂ and 116 ♀	77/116 (66.4%)	59/77 (76.6%)	6/59 (10.2%)	ND
39 ♂ and 78 ♀	61/78 (78.2%)	ND	ND	30/61 (49.2%)

^1^ Surviving females were sampled 7 days after the initial cohabitation.

**Table 3:** Venereal transmission of VSV from venereally infected *Culicoides* males (4 days post-mating) to naïve females as detected by whole midge RT-qPCR.

Initial Number of Midges	Surviving ♀ (%) ^1^	VSV RT-qPCR+ Whole ♀ (%)
100 ♂ and 200 ♀	84/200 (42%)	8/84 (9.5%)

^1^ Surviving females were sampled 7 days after the initial cohabitation.

**Table 4:** Organs with positive fluorescent puncta staining of intrathoracically inoculated *C. sonorensis* midges 4 dpi.

Males		Females	
Testes	Accessory Glands	Ovaries	Spermatheca
15/17 (88.2%)	7/17 (41.2%)	11/12 (91.7%)	0/12 (0%)

**Table 5:** Organs with VSV-positive staining of sections of orally infected *C. sonorensis* females (8–13 dpf).

Ovaries	Oviduct	Spermathecal Duct	Gonotreme	Gonopore
7/19 (36.8%)	3/19 (16.8%)	5/19 (26.3%)	6/19 (31.6%)	11/19 (57.8%)
